# The Minnesota’s Medical Monthly

**Published:** 1887-06

**Authors:** 


The Minnesota Medical Monthly comes to us regularly, and is a bright, interesting journal; among other good matter in its May issue we noted especially two articles by Drs. W. E. Leonard and H. C. Leonard.

